# The C-MAC video laryngoscope helps presbyopic anesthetists to overcome difficulty in neonatal and infantile intubation: a randomized controlled trial

**DOI:** 10.1186/s12871-024-02841-x

**Published:** 2025-01-10

**Authors:** Ashraf E. Abdalla, Mohsen M. Eissa, Mohamed R. Elbasyouny, Mahmoud R. Zomra, Ahmed M. Elnaggar, Mahmoud M. Elsayed

**Affiliations:** Department of Anesthesia, ICU & Pain, Faculty of Medicine (Boys), Al-Azhar University, Cairo, Egypt

**Keywords:** C-MAC video-laparoscope; infants, Neonates, Presbyopic anesthetists, 30-s success intubation rate

## Abstract

**Background:**

Endotracheal intubation (ETI) is a life-saving procedure that must be accurately carried on to guard against complications. Presbyopia leads to difficulty in viewing close objects and may obstacle proper intubation even with the best hands.

**Purpose:**

This study supposed that the use of video-laryngoscope (VL) may provide better intubation conditions for presbyopic anesthetists and targets to evaluate the neonates and infants’ intubation success rates (ISR) by anesthetists aged ≥ 45 years using the C-MAC VL compared to the standard laryngoscope (SL).

**Methods:**

Thirty-one neonates with an age of 18.2 ± 5.2 days and a body weight of 4.5 ± 0.3 kg and 103 infants aged 8.6 ± 1 months and weighing 9.4 ± 1.5 kg were randomly categorized into the SL group that received ETI using the SL and the VL group had intubated using the C-MAC^®^ (Karl Storz, Germany) VL with the standard Miller blade and flexible Stylet (2 mm PORTEX^®^ stylet; Smiths Medical International Ltd., UK) to strengthen the endotracheal tube (ETT) and adjust its curvature as C-shaped. The study outcomes included the frequency of successful intubation and the number of intubation attempts.

**Results:**

The ISR was significantly (*P* < 0.001) higher with significantly (*P* = 0.0037) lower frequency of using assistance maneuvers with VL. The mean score of the anesthetist’s difficulty rating was significantly (*P* < 0.001) higher with SL (2.7 ± 2) than with VL (1.27 ± 1.27). Times for the full intubation process were significantly (*P* < 0.001) shorter with VL than SL. The 1st attempt success rate was significantly (*P* = 0.0195) higher with VL than SL (86.6% vs. 67.2%). The frequency of maneuver-related complications was insignificantly (*P* = 0.116) reduced with the use of VL (4.5%) than with SL (12%). The ISR showed a negative significant correlation (*r*=−0.973, *P* = 0.005) with the anesthetist’s age.

**Conclusion:**

Neonatal and infantile intubation using VL is feasible and easy to handle by aged anesthetists and allows higher ISR and 1st attempt rate with minimal need for external assistant maneuvers and maneuver-related complications. VL might be more appropriate for the presbyopic pediatric anesthetists than the SL.

**Limitations:**

The limitations of the study are the small sample size of anesthetists and the use of one type of VLs.

## Introduction

Securing an airway is a life-saving procedure for the provision of lungs’ ventilation and proper blood oxygenation, however, adverse and critical events are not uncommon during airway management, particularly in neonates and infants [1].

Efficient neonatal airway management is challenging even in the most experienced hands and the prevalence of difficult intubation in pediatric anesthesia varies greatly on a wide range [2].

Neonatal intubation is a life-saving procedure, which requires a skilled operator but still may cause direct tissue trauma and precipitate adverse reactions. However, intubation with video-laryngoscope (VL) requires less force than with a direct laryngoscope to minimize the possibility of these adverse events [3].

The recent Brazilian recommendations for the management of pediatric difficult airways included proper assessment, preparation, positioning, pre-oxygenation, minimizing trauma, maintenance of arterial oxygenation, and the implementation of advanced tools such as VL, flexible intubating bronchoscopy, and supraglottic devices [4]. The recent British recommendations also advised the use of VL with an age-adapted standard blade as the first choice for tracheal intubation and the use of a stylet to reinforce and pre-shape tracheal tubes in case of the use of hyper-angulated VL blades [5].

Considering the recent interest in assessing the performance of various VLs in pediatric anesthesia, the C-MAC^®^ (Karl Storz, Germany) VL with standard Miller blade sizes #0 and #1, is widely used in neonates and infants [6] for its provision of superior-quality glottis view in comparison to the McGrathTM MAC size #1 blade and direct laryngoscopy [7].

Presbyopia is defined as a disordered eye adjustment function that affects middle-aged people leading to difficulty in viewing close objects and is corrected with a magnifying lens [8]. Earlier studies documented that presbyopic anesthetists find difficulty when trying to view a patient’s larynx at a close distance and this difficulty is surely magnified on dealing with the intubation of neonates and children [9–11].

### Hypothesis

This study speculated that using VLs for intubating neonates may facilitate the intubation process, allow easier procedures for aged presbyopic anesthetists, and thus significantly improve their performance in dealing with neonatal intubation.

### Objectives

Evaluation of the success rates of anesthetists older than 45 years for intubating neonates and infants using the C-MAC VL in comparison to the use of the conventional standard laryngoscope (SL).

### Design

Prospective randomized controlled interventional study.

### Setting

Department of Anesthesia, Pain and ICU, Faculty of Medicine, Al-Azhar University.

### Sample size

The sample size was calculated using the G*Power (Version 3.1.9.2) [12], to provide a study power of 80% (z = 1.28) using α-error 5%, and considering the effect size (ῥ) of 0.20, 47 patients per group was defined as the suitable number to ensure the certainty of the null hypothesis, which was suggested to be a significant difference in the frequency of the success of the 1st attempt for tracheal intubation and to guard against dropouts each group started with 50 patients per group.

### Study protocol

The study protocol entailed the collection of demographic and clinical data of pediatric patients who were planned to undergo surgical procedures under general inhalational anesthesia with endotracheal intubation through the duration from Jan 2021 to Jan 2024. These patients were randomly divided into two groups according to the procedure of tracheal intubation using SL (Group-SL) or VL (Group-VL). All anesthetists in charge were experienced in pediatric anesthesia and were older than 40 years; each anesthetist had to manage at least 10 patients from each group.

### Preoperative assessment

The collected data for anesthetists included age and the use of glasses or lenses and for patients included age, weight, gender, ASA grade and indication for surgery.

1. Preoperative determination of the external airway measurements that were previously documented to potentially predict potentially difficult airways. These measures included the Sternomental (SMD) and thyromental (TMD) distances were calculated according to the documented age-dependent equations as 8.82 + 0.51 × Age and 5.03 + 0.23 × Age, respectively [13].
2. Evaluation of physiognomic features associated with difficult laryngoscopy using the Mallampati score: Class 1: Faucial pillars, soft palate and uvula could be visualized; Class 2: Faucial pillars and soft palate could be visualized, but uvula was masked by the base of the tongue; Class III: Soft palate, the base of uvula visible and Class IV: Soft palate not visible at all [14].
3. Evaluation of the laryngoscope views using the direct laryngoscopy for cases of SL group using the modified Cormack–Lehane classification system as Grade-I on visualization of the entire laryngeal aperture; Grade-IIa on partial viewing of the glottis & Grade-IIb if only the arytenoid cartilage or the posterior extremity of the glottis is seen; Grade-III on visualization of only the epiglottis and Grade-IV when the soft palate is the only visualized part but both of the epiglottis and the glottis are not seen [15]. Patients of the VL group took advantage of LV; the situation of the lens or camera near the tip of the laryngoscope blade and the larynx, with an angle of view of 50–60°, which almost certainly allows better view than with direct laryngoscopy [16].

### Exclusion criteria

Patients older than 12 months, patients with Mallampati score of 3 or 4, patients who had abnormal airways, obstructive sleep apnea, manifestations of upper respiratory tract infection, or uncompensated cardiopulmonary diseases, and patients with ASA grade > III or chromosomal abnormalities were excluded from this study.

### Inclusion criteria

Patients younger than 12 months, free of exclusion criteria, and scheduled for surgical procedures under general inhalation anesthesia were included in the study.

### Randomization and grouping

The randomization process was conveyed by an assistant who was blinded about the significance of the used letters. Patients were randomly divided into two groups using the random block sizes of 2 and 4 by 1:1 allocation computer randomization method (Excel 2007, Microsoft, Redmond, WA, USA) to generate the sequence for the distribution of patients between both groups. The generated sequences were printed on cards, and enveloped in opaque envelopes. Parents were asked to choose a card and propose the card to the anesthetist in charge who was blinded about the randomization process and the type of laryngoscope to be used. Patients were categorized into two groups: the SL group, patients who received intubation using the standard laryngoscope, and the VL group patients who had intubated using the C-MAC^®^ (Karl Storz, Germany) VL with the standard Miller blade. The cards were equally divided between the sharing anesthetists to equalize the results between them irrespective of their age or the mode for error correction; glasses or lenses.

### Preparation for Intubation procedures

Endotracheal intubation was performed using the microcuff pediatric endotracheal tubes (ETT). The choice of tube size was age-adjusted according to the outer-to-inner diameter ratio as follows: tubes with internal diameter (ID) of 3.0 mm were applied for patients aged < 6 months and tubes with ID 3.5 mm for patients aged 6-<18 months [17]. Flexible Stylet (2 mm PORTEX^®^ stylet; Smiths Medical International Ltd., UK) was used to strengthen the ETT and adjust its curvature as a C-shaped curvature.

### Anesthetic procedure

Once the patient was admitted to the operative room, non-invasive monitoring was started, the patient was pre-oxygenated using facial mask ventilation, and open venous access was assured. Anesthesia was induced by a sevoflurane mask and intravenous injection of fentanyl 1–2 µg/kg with rocuronium bromide (0.5–1.0 mg/kg) as a muscle relaxant. Then, a facial mask was used for ventilation with sevoflurane and 100% oxygen. With the patient’s head in the neutral position, the age-adjusted ETT with a loaded stylet was inserted after securing the best glottic view using either the standard laryngoscope or C-MAC^®^ VL with a Miller blade size #0 or #1. The choice of the blade was age-dependent with blade #0 for neonates and #1 for infants as previously documented [6, 18]. During each attempt of the endotracheal tubal insertion, if the operator failed to advance the ETT through the glottis or the patient’s oxygen saturation decreased to < 95%, the tube was withdrawn and manual ventilation with 100% oxygen was initiated to restore oxygen saturation to 98–100% before the further intubation attempt and the attempt was considered failed.

### Evaluation tools

1. Intubation times included: time to best glottis view (T0), time to approach ETT tip to glottis after glottis visualization (T1), and time to advance ETT cuff fully through the glottis (T2). Then, tube handling time was calculated as the sum of T1 and T2, and total intubation time which is the sum of tube handling time plus T0.
2. The trial success rate was defined as the number of succeeded intubations during the first attempt. The number of attempts to achieve intubation per anesthetist was calculated and the relation between the anesthetist’s age and success rate.
3. The intubation difficulty scale (IDS): Each anesthetist has to rate the intubation difficulty using either laryngoscope on a 10-point numerical rating scale with 0 indicating extremely easy and 10 indicating extreme difficulties [19].
4. Patients were observed after extubation for dental injury, laryngeal injury and were transferred to the post-anesthetic care unit then were discharged to the corresponding ward according to the type of surgery and were re-evaluated at four hours after surgery for the development of postoperative (PO) sore throat that was predicted if the patient was crying, restless, or agitated [20].

### The study outcomes

1. The primary outcome is the frequency of successful intubation.
2. The secondary outcomes included.

- The frequency of successful intubation within 30 s (30-s success rate; 30-s SR).
- The number of intubation attempts.
- The frequency of the need for external laryngeal manipulation, head extension, or stylet curvature change to achieve intubation.
- The anesthetists subjective rating of intubation difficulty.

### Statistical analysis

The Kolmogorov-Smimov test of normality and the normal Q-Q plots were used to test the data normality. The data are presented as mean, standard deviation, numbers, and percentages. The intergroup differences for data presented as mean and standard deviation (SD) were compared using a one-way ANOVA test with Tukey HSD, while data presented as percentages were compared using the Chi-square test. The correlation between the number of successful 1st attempts and the anesthetists’ age was evaluated using Pearson’s correlation analysis and presented as Pearson’s correlation coefficient. Statistical analyses were conveyed by the IBM^®^ SPSS^®^ Statistics software (Ver. 27, 2020; IBM Corporation; Armonk, USA). The significance of the analysis was evaluated at the cutoff point of P less than 0.05.

## Results

**Fig. 1:**
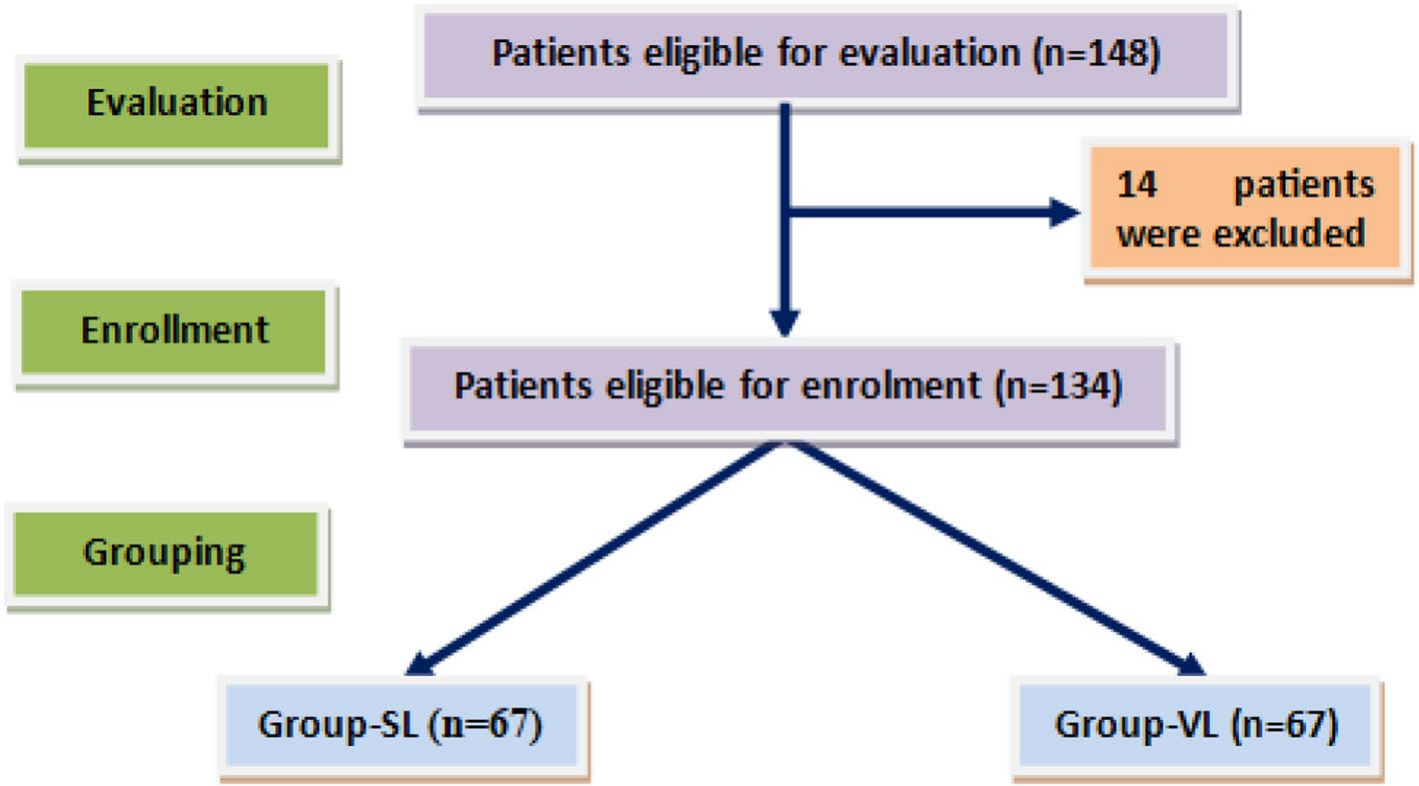
The study flowchart

Preoperative assessment excluded 14 patients; 5 patients had a Mallampati score of ≥ 3, 3 patients had manifestations of obstructive sleep apnea, two patients had a cleft palate, another two patients had a cleft lip and one patient had a thyroglossal fistula (Fig. 1). One-hundred thirty-four patients; 31 neonates with a mean age of 18.2 ± 5.2 days and a mean weight was 4.5 ± 0.3 kg and 103 infants had a mean age of 8.6 months (± 1) and mean body weight of 9.4 ± 1.5 kg. Patients were randomly divided into two study groups and showed insignificant differences as regards enrolment data (Table 1). The enrolled anesthetists had a mean age of 54.6 ± 5.5; range: 49–63 years. Three anesthetists were glass users, while two were lens users.

**Table 1:** Enrolment data of patients of both groups

Data Group			SL (*n* = 67)	VL (*n* = 67)	*P*-value
Age data	Distribution	Neonates	14 (20.9%)	17 (25.4%)	0.539
		Infants	53 (79.1%)	50 (74.6%)	
	Mean (± SD)	Neonates (days)	19 ± 4	18 ± 4.5	0.523
		Infants (month)	8.6 ± 1.1	6.7 ± 0.8	0.543
Gender	Male		39 (58.2%)	44 (65.7%)	0.373
	Female		28 (41.8%)	23 (34.3%)	
Body weight (kg)		Neonates	4.4 ± 0.25	4.5 ± 0.32	0.346
		Infants	9.5 ± 1.6	9.3 ± 1.2	0.412
ASA grade	I		53 (79.1%)	57 (85.1%)	0.367
	II		14 (20.9%)	10 (14.9%)	
Type of surgery	General surgery		25 (37.4%)	22 (32.9%)	0.945
	Urological surgery		11 (16.4%)	10 (14.9%)	
	Cardiac surgery		9 (13.4%)	11 (16.4%)	
	Chest surgery		6 (9%)	8 (11.9%)	
	orthopedic		9 (13.4%)	10 (14.9%)	
	Otorhinolaryngology		7 (10.4%)	6 (9%)	
Mallampati score	Class I		46 (68.7%)	51 (76.1%)	0.333
	Class II		21 (31.3%)	16 (23.9%)	
C-L score	Class I		65 (97%)	61 (91%)	0.145
	Class II		2 (3%)	6 (9%)	
SMD (cm)			13.21 ± 0.55	13.26 ± 0.41	0.543
TMD (cm)			7.01 ± 0.25	7.03 ± 0.18	0.709

*SL *Standard laryngoscope, *VL *Videolaryngoscope, *C-L score* modified Cormack–Lehane score, *SMD* Sternomental distance, *TMD* Thyromental distance, P indicates the significance of intergroup differences at *P* < 0.05

**Fig. 2:**
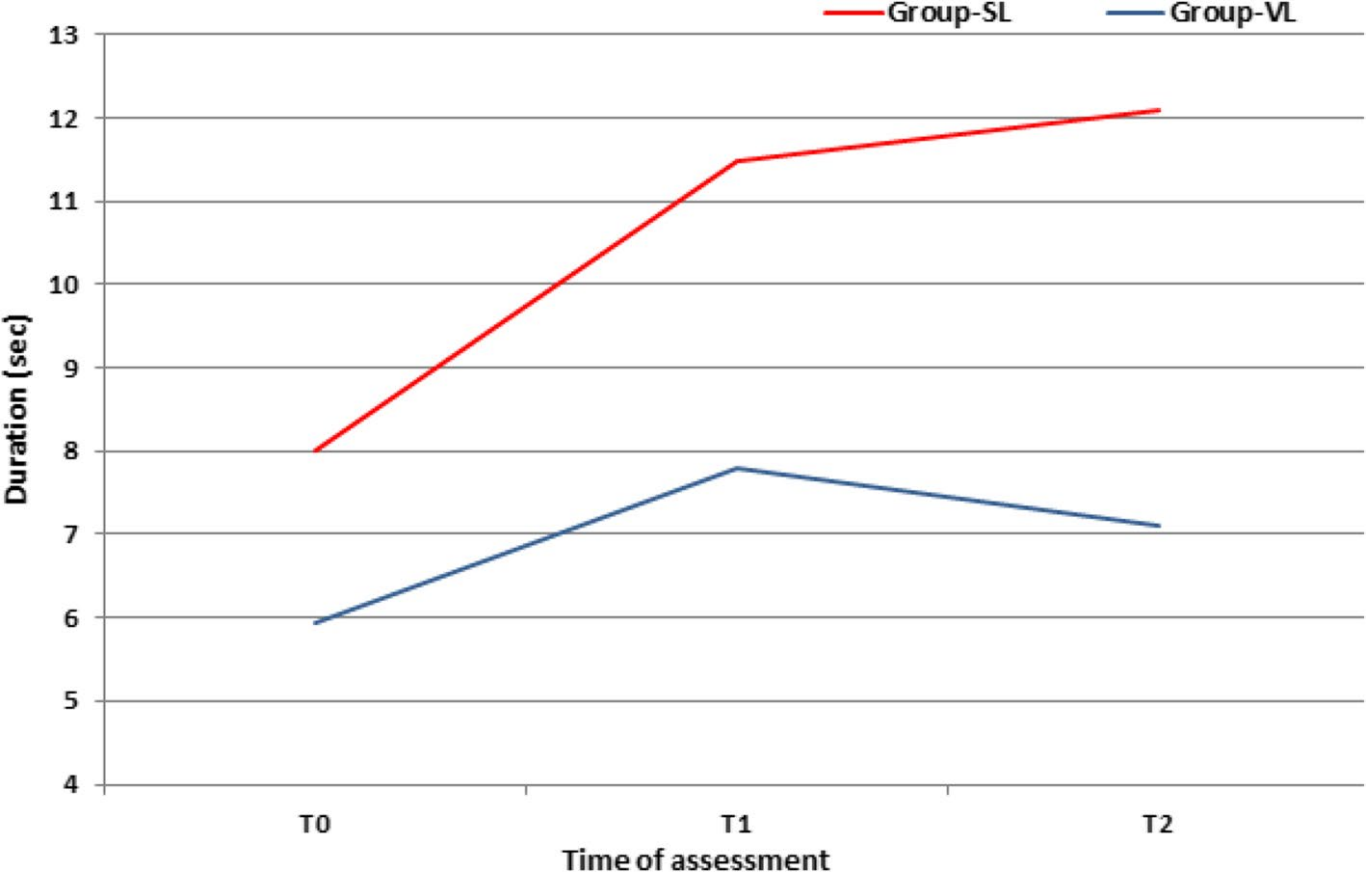
Differential times till fulfillment of the intubation process

**Fig. 3:**
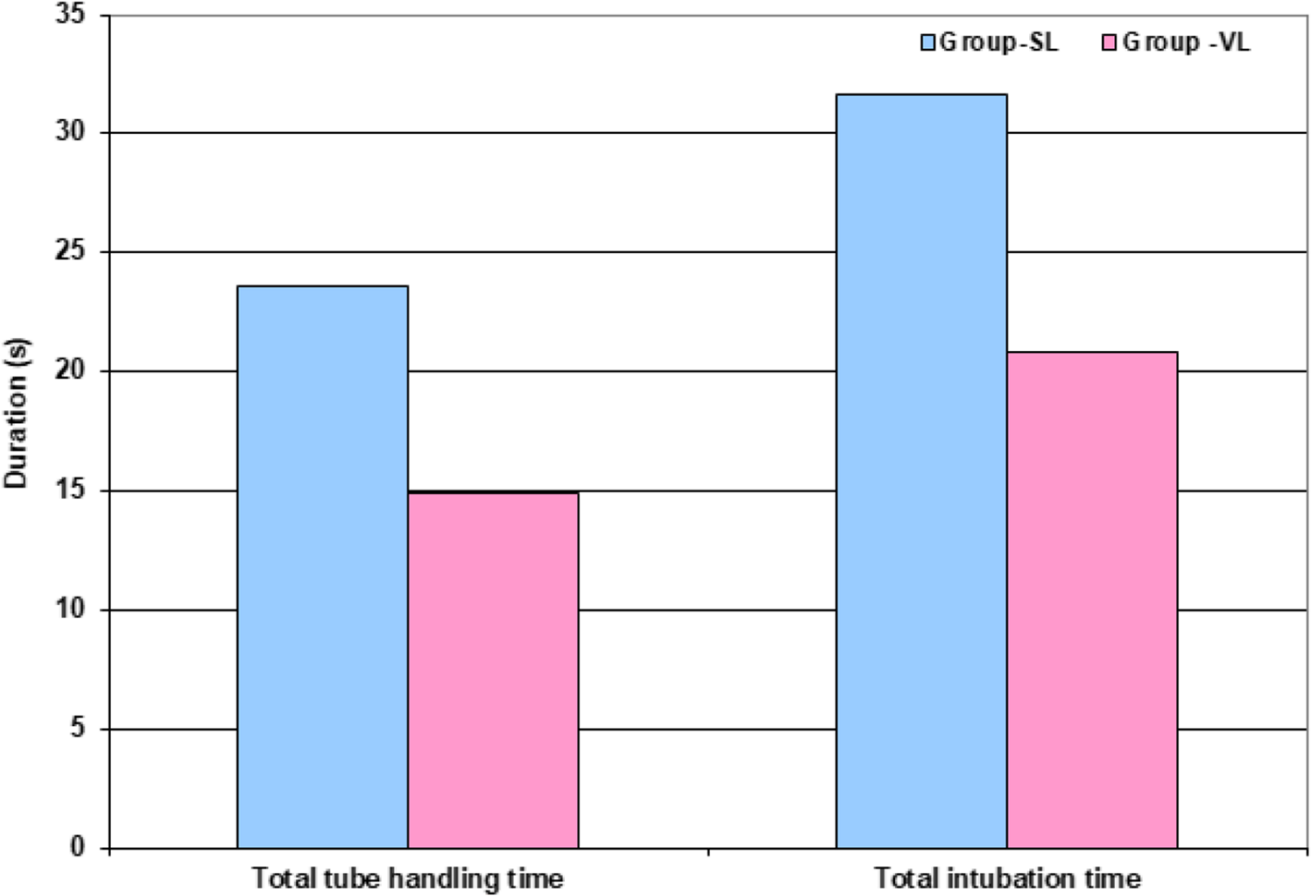
Collective time consumed till fulfillment of the intubation process

The use of VL allowed more rapid tube handling as manifested by the significantly (*P* < 0.001) shorter time to best glottis view, time to approach of ETT to glottis after its visualization, and time till full advancement of the ETT cuff through the glottis in comparison to time consumed by the SL (Fig. 2). Collectively, total tube handling time and total time consumed for full intubation process was significantly (*P* < 0.001) shorter with the use of VL than with SL (Table 2; Fig. 3).

**Table 2:** Time consumed by anesthetists for the completion of the intubation process using SL and VL

Data Group	SL (*n* = 67)	VL (*n* = 67)	*P*-value
Time to best glottis view (T0) (s)	8 ± 2.7 (4–14)	5.9 ± 2.2 (2–10)	< 0.001
Time to approach ETT tip to glottis after glottis visualization (T1) (s)	11.5 ± 1.75 (8–16)	7.8 ± 3.5 (3–16)	< 0.001
Time to full advancement of the ETT cuff through the glottis (T2) (s)	12.1 ± 6.8 (4–27)	7.1 ± 4.3 (1–18)	< 0.001
Tube handling time (s)	23.6 ± 7.5 (12–40)	14.9 ± 7 (4–33)	< 0.001
Total intubation time (s)	31.6 ± 8.1 (16–49)	20.8 ± 8.4 (8–40)	< 0.001

Data are presented as mean, standard deviation and ranges in parenthesis; *SL* Standard laryngoscope, *VL* Video laryngoscopy, *ETT* Endotracheal tube, Tube handling time equals the sum of T1 and T2; Total intubation time equals the sum of T0, T1 and T2; P indicates the significance of intergroup differences at *P* < 0.05

Tracheal intubation was accomplished within 30 s in 91 patients for a successful within 30-sec endotracheal tubal insertion rate of 67.9%. The frequency of successful endotracheal tubal insertion within 30-sec was significantly (*P* < 0.001) higher with VL than with SL (86.6% vs. 49.3%). Assistance maneuvers were required during the endotracheal tubal insertion in 61 patients (45.5%) including 41 patients (61.2%) in the SL group and 20 patients (29.9%) in the VL group with significantly (*P* = 0.0037) lower frequency among patients of VL group. The head extension and external laryngeal manipulation were the most frequently applied maneuvers with SL to aid in straightening the passage for tube insertion with minimal difficulty and pushing power to minimize the possibility of trauma. The frequency of maneuver-related complications was comparable (*P* = 0.116) but in favor of VL (12% vs. 4.5%). The mean value of the anesthetist’s difficulty rating score was significantly (*P* < 0.001) higher with SL than with VL with significantly (*P* < 0.001) lower frequencies of low rating scores for SL than VL (Table 3).

**Table 3:** Trial outcomes

Data Group				SL (*n* = 67)	VL (*n* = 67)	*P*-value
Success rate within 30-sec				33 (49.3%)	58 (86.6%)	< 0.001
Assistance maneuvers	No			26 (38.8%)	47 (70.1%)	0.0037
	External laryngeal manipulation			18 (26.9%)	10 (14.9%)	
	Head extension			13 (19.4%)	6 (9%)	
	Stylet curvature change			10 (14.9%)	4 (6%)	
Anesthetists’ subjective rating of intubation difficulty		Mean		2.7 ± 2	1.27 ± 1.27	< 0.001
		Rating score	0	14 (20.9%)	22 (32.8%)	< 0.001
			1	9 (13.4%)	21 (31.3%)	
			2	10 (14.9%)	15 (22.4%)	
			3	6 (9%)	4 (6%)	
			4	11 (16.4%)	3 (4.5%)	
			5	11 (16.4%)	2 (3%)	
			6	6 (9%)	0	
Maneuver-related complications		Dental injury		2 (3%)	1 (1.5%)	0.559
		Laryngeal injury		1 (1.5%)	0	0.816
		Postoperative sore throat		5 (7.5%)	2 (3%)	0.244
		Total		8 (12%)	3 (4.5%)	0.116

*SL* Standard laryngoscope, *VL* Videolaryngoscope, *ETT* Endotracheal tube, Tube handling time equals to the sum of T1 and T2; Total intubation time equals to the sum of T0, T1 and T2; P indicates the significance of intergroup differences at *P* < 0.05

**Fig. 4:**
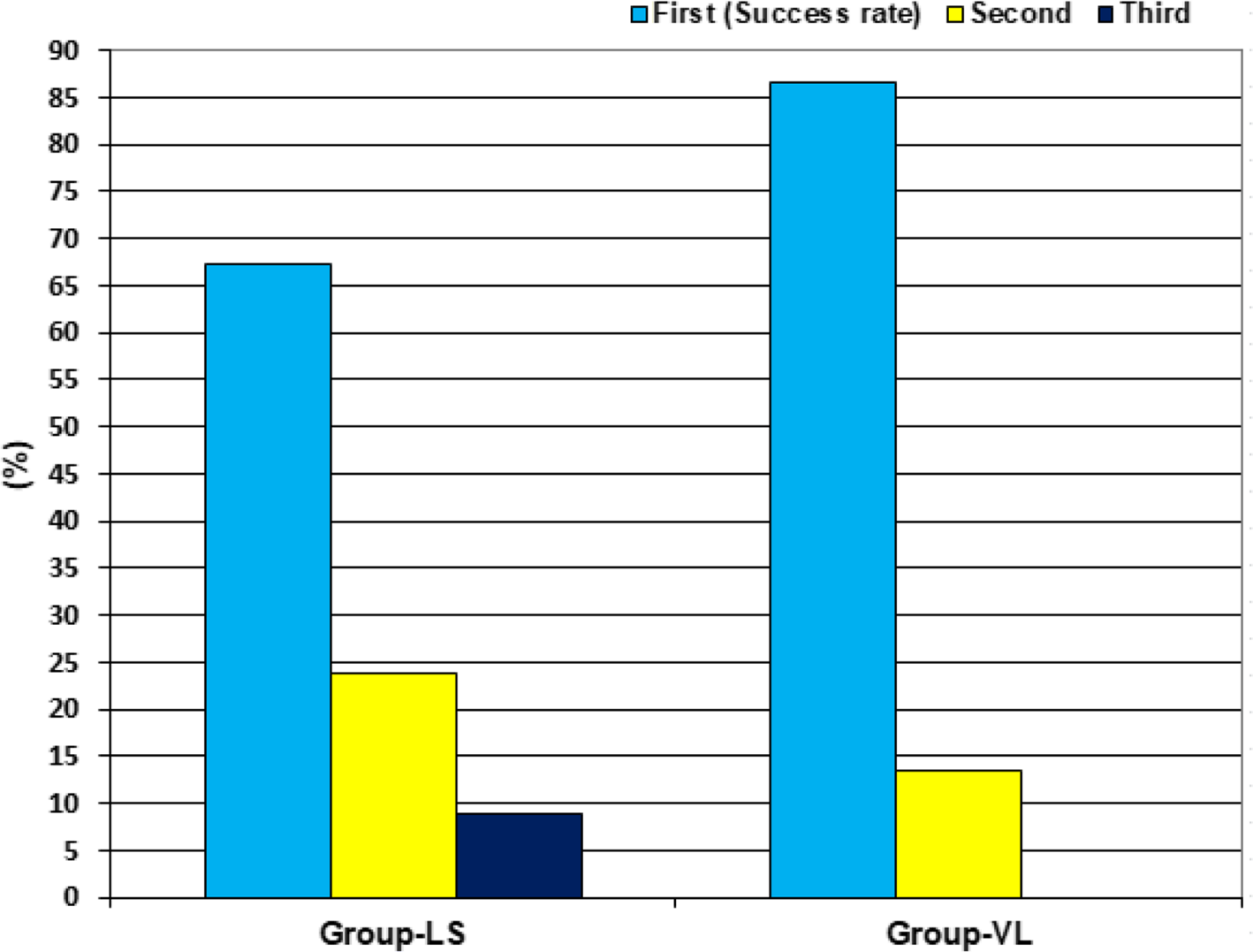
Patients' distribution according to attempts of intubation

**Fig. 5:**
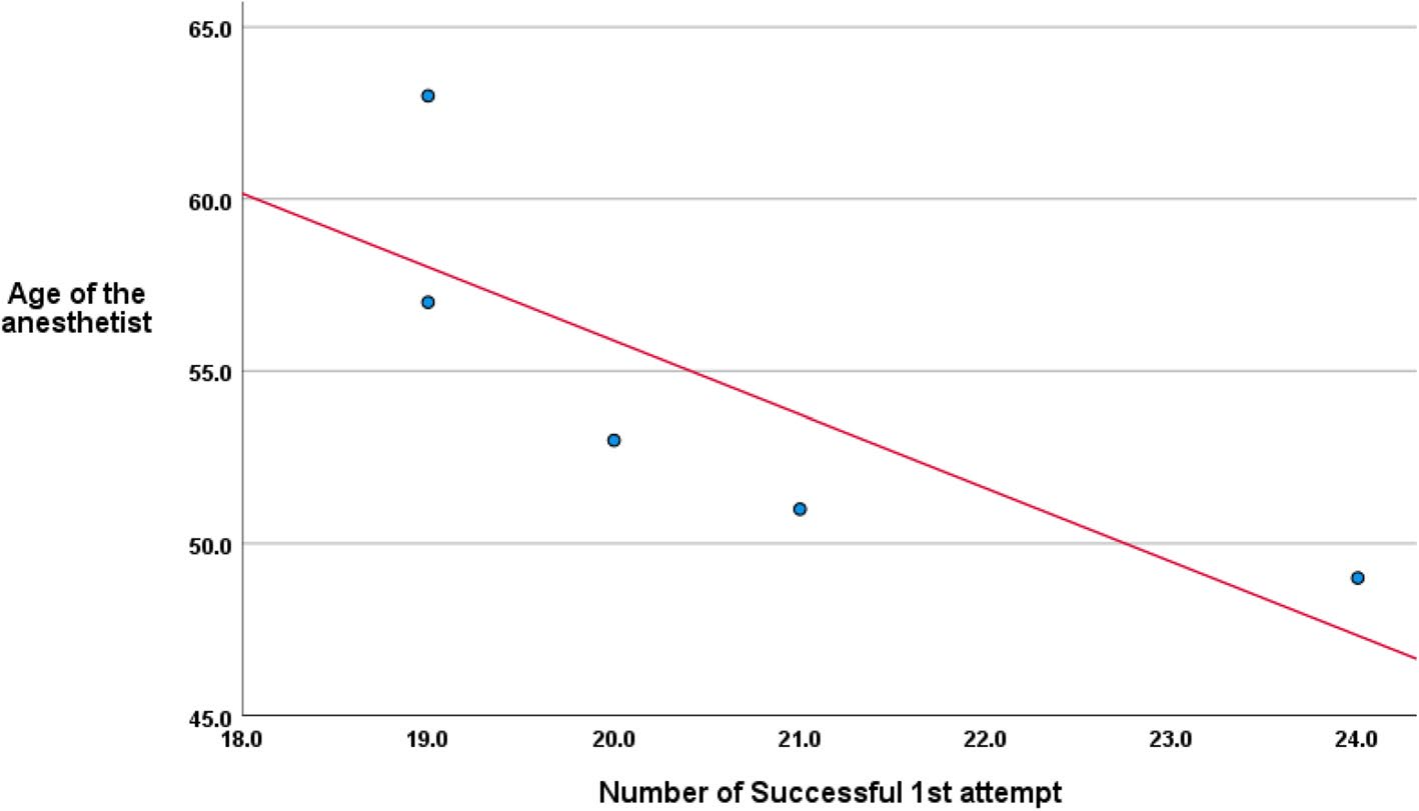
Correlation between the number of successful 1^st^ attempts and the age of the anesthetist

 The frequency of success of tube insertion for each anesthetist was insignificantly higher with the use of VL than with SL. However, the total success rate with VL was significantly (*P* = 0.0195) higher than with SL (86.6% vs. 67.2%). Moreover, no anesthetist required to perform three attempts with VL, while with SL 4 anesthetists (80%) performed three attempts (Table 4; Fig. 4). The success rate for the endotracheal tubal insertion showed an intimate inverse relation (*r*=−0.973, *P* = 0.005) with anesthetist’s age (Fig. 5).

**Table 4:** The success rate of the endotracheal tubal insertion for each anesthetist

	Attempts	Anesthetist No.					Total
		1	2	3	4	5	
SL	One	11 (78.6%)	8 (61.5%)	8 (57.2%)	9 (69.2%)	9 (69.2%)	45 (67.2%)
	Two	2 (14.3%)	3 (23.1%)	5 (35.7%)	2 (15.4%)	4 (30.8%)	16 (23.8%)
	Three	1 (7.1%)	2 (15.4%)	1 (7.1%)	2 (15.4%)	0	6 (9%)
	Total	14 (20.9%)	13 (19.4%)	14 (20.9%)	13 (19.4%)	13 (19.4%)	67 (100%)
VL	One	13 (92.9%)	11 (84.6%)	11 (78.6%)	11 (84.6%)	12 (92.3%)	58 (86.6%)
	Two	1 (7.1%)	2 (15.4%)	3 (21.4%)	2 (15.4%)	1 (7.7%)	9 (13.4%)
	Total	14 (20.9%)	13 (19.4%)	14 (20.9%)	13 (19.4%)	13 (19.4%)	67 (100%)
P-value		0.280	0.185	0.225	0.352	0.135	0.0195
Both	One	24 (85.7%)	19 (73.1%)	19 (67.8%)	20 (76.9%)	21 (80.8%)	103 (76.9%)
	Two	3 (10.7%)	5 (19.2%)	8 (28.6%)	4 (15.4%)	5 (19.2%)	25 (18.6%)
	Three	1 (3.6%)	2 (7.7%)	1 (3.6%)	2 (7.7%)	0	6 (4.5%)
	Total	28 (20.9%)	26 (19.4%)	28 (20.9%)	26 (19.4%)	26 (19.4%)	134 (100%)

## Discussion

The use of VL facilitated the intubation of infants younger than 12 months in the hand of all of the enrolled anesthetists, irrespective of their age, visual acuity, or the use of error correcting device. The reported 30-s success rate (30-s SR) was significantly higher with VL than with SL and showed a negative significant relation to anesthetist’s age. These findings assured the study null hypothesis that the use of VL might improve the performance of old-aged anesthetists and mitigate the impact of presbyopia on times of tube handling. The negative relation between an anesthetist’s age and success rate might be attributed to the fact that old-aged anesthetists were mostly accustomed to using the SL and were less trained to use the VL.

These findings go in hand with Lambert et al. [21], who found Tansen VL showed intubation performance superior to Macintosh laryngoscope and non-inferior to the Pentax-AWS VL as regards the intubation success rate, grade of laryngeal view and time to intubation. Also, Gupta et al. [7] compared the intubation data with the use of C-MAC Miller or McGrath MAC for infants receiving general anesthesia and found the C-MAC Miller blade provided superior glottic views, but showed similar intubation timings, success rates, and intubation difficulty score as compared to McGrath MAC in neonates and infants. Additionally, Pantazopoulos et al. [22], in manikin study, compared intubation using the VL or conventional Macintosh laryngoscope by novice physicians and detected significantly shorter insertion time on the use of VL with significantly higher first-pass success rate. Moreover, Kim et al. [23], detected significantly higher percentage of glottic opening and significantly lower modified Cormack-Lehane (C&L) grade and score on intubation difficulty scale with C-MAC D-blade VL compared to McCoy laryngoscope, but total time taken for the intubation process, malposition status, hemodynamic parameters, or scoring on the visual analog scale for postoperative sore throat were comparable.

In line with the obtained results and the study null hypothesis, Ratajczyk et al. [24], compared the performance of experienced and inexperienced anesthetists in intubation using the intubrite VL versus the SL and found VL provided better working conditions and reimbursed for deficiencies in successful tracheal intubation by inexperienced participants both in normal and difficult intubation environment, but its effectiveness was higher in hand of the experienced anesthetists, irrespective of the intubation environment.

Also, Prekker et al. [25], analyzed the intubation process using direct versus VL and found VL improved the grade of view with 1st attempt success rate of 83.2% on using VL versus 72.2% with direct laryngoscope and concluded that the use of VL was associated with better view of the vocal cords and higher probability of successfully intubating the trachea when the view of the vocal cords was incomplete.

Moreover, Park et al. [26] compared the tube-handling time between a C-curved and hockey stick-shaped stylet in infants and neonates using the C-MAC^®^ VL Miller blade and found Tube insertion time and total intubation duration were significantly shorter in group C than in group H with significantly higher intubation success within 30 s than group H (87.7% vs. 69.8%), and concluded that C-curve ETT shape may reduce tube handling time than the hockey stick-shaped tube during intubation using a C-MAC^®^ VL Miller blade in neonates and infants.

Contrary to the obtained results, Küçükosman et al. [27], using the direct lifting method of the epiglottis compared Miller laryngoscope and McGrath-MAC VL detected insignificant differences as regards hemodynamic responses, intubation time, number of attempts, duration of view of the glottis opening, and the need for external laryngeal pressure with similar effectiveness in terms of percentage of glottic opening and C&L score when used with the direct lifting method of the epiglottis. However, Küçükosman et al. [27], did not comment on the age and experiences of the anesthetists included in the intubation process, but the insignificant differences point to equal experience of the anesthetists in change with both laryngoscopes.

Recently, Kamal et al. [28], found the use of the C-MAC VL size-2 D-blade in comparison to McCoy size-2 laryngoscope in children provided faster and better glottic visualization with shorter time to achieve glottic view but with similar intubation difficulty and insignificant differences concerning the total duration of intubation and attributed this to the pronounced curvature of the D-blade. Also, Park et al. [29] documented that during intubation of neonates and infants using a C-MAC^®^ video laryngoscope Miller blade modification of the ETT shape into a C-curve may reduce tube handling time compared to the conventional hockey stick-shaped tube and laryngoscope operators rated intubation as easier when provided with a C-curved stylet.

In support of the efficacy and safety of the C-MAC VL, Paik & Park [30] in manikin with cervical immobilization found the C-MAC D-Blade VL causes less upper cervical spine motion than the Macintosh laryngoscope during tracheal intubation. As regards, forces applied during laryngoscopy multiple studies using manikin documented that VLs are advantageous for the provision of significantly decreased force exerted on maxillary incisors and thus might reduce the risk for dental injury in clinical settings and this was evident with hyper-angulated VL [3, 31–33]. Furthermore, Koch et al. [34], found about 45% of the emergency medical service physicians use VL for pre-hospital endotracheal intubation and showed significantly higher compliance with CMAC^®^ or McGrath^®^.

Regrettably, the literature review failed to find recent studies dealing with a similar topic apart from the earlier study [9] that suggested the use of the Airtraq for its provision of magnified observation, which helps the presbyopic anesthetists to read a Landolt ring mark placed in front of the vocal cords faster than the direct observation using Macintosh laryngoscope.

## Conclusion

The use of VL for endotracheal intubation of infants aged ≤ 12-m is feasible and easy to handle by aged anesthetists and allowed high 30-s success rate and high 1st attempt rate with minimal need for external assistant maneuvers and maneuver-related complication. VL might be considered to be appropriate for aged presbyopic anesthetists especially those who had error of refractions.

### Limitation

The small sample size of anesthetists and the use of one type of VLs are the study limitations.

### Recommendations

Multicenter wider-scale studies including a large number of anesthetists and instituting teaching seminars and training programs to allow old anesthetists be familiar with the use of VLS. Also, comparative studies for various types of VLs are mandatory to establish the obtained results and verify the advantages and disadvantages of each type with special regard to the cost-benefits of the use of VLs. Moreover, training campaigns with invitations of the manufacturers to present their most recent advent in VLs to the anesthetists.

## Data Availability

No datasets were generated or analysed during the current study.
